# Innate, High Tolerance to Zinc and Lead in Violets Confirmed at the Suspended Cell Level

**DOI:** 10.3390/cells11152355

**Published:** 2022-07-31

**Authors:** Szymon Miszczak, Klaudia Sychta, Sławomir Dresler, Agnieszka Kurdziel, Agnieszka Hanaka, Aneta Słomka

**Affiliations:** 1 Department of Plant Cytology and Embryology, Institute of Botany, Faculty of Biology, Jagiellonian University in Kraków, Gronostajowa 9 Str., 30-387 Cracow, Poland; szymon.miszczak@student.uj.edu.pl (S.M.); klaudia.sychta@uj.edu.pl (K.S.); agnieszka.kurdziel@doctoral.uj.edu.pl (A.K.); 2 Department of Analytical Chemistry, Medical University of Lublin, Chodźki 4a Str., 20-093 Lublin, Poland; slawomir.dresler@umlub.pl; 3 Department of Plant Physiology and Biophysics, Institute of Biological Sciences, Faculty of Biology and Biotechnology, Maria Curie-Skłodowska University, Akademicka 19 Str., 20-033 Lublin, Poland; agnieszka.hanaka@mail.umcs.pl

**Keywords:** facultative metallophyte, tolerance, heavy metal, suspended cells

## Abstract

Many species of the *Viola* L. genus (violets) colonize areas with high concentrations of trace elements in the soil, e.g., nickel, cadmium, zinc, and lead. Although tolerance to heavy metals is a common phenomenon in violets, it is not clear whether this is the result of gradual microevolutionary processes as a part of the adaptation to the specific conditions, or whether the tolerance was inherited from the ancestor(s). We developed cell suspension cultures of five plant species: two non-metallophytes—*Arabidopsis thaliana* (Col-0) and *Viola* · *wittrockiana*, and three metallophytes—*V. philippica*, *V. tricolor,* and *Silene vulgaris* subsp. *humilis* for tolerance tests. The aim of the study was to measure the level of tolerance of violets in comparison with species from the other genera to verify the hypothesis of the high, innate tolerance of the former. We measured cell viability, non-enzymatic antioxidant content, and the accumulation of heavy metals after cell treatment with Zn or Pb. The results indicate they are innate and independent on the ecological status (metallophyte vs. non-metallophyte) and high in comparison with other species tolerance to Zn and Pb in violets. Viability of the cells after Zn and Pb (1000 μM) exposure for 72 h was the highest in violets. Antioxidant content, after heavy metal treatment, increased significantly, particularly in metallophyte violets, indicating their high responsivity to metals. In all species, lead was detected in the protoplasm of the cells, not in the vacuole or cell wall. All violets were characterized by the accumulation capacity of lead. Here, we clearly show that the physiological and biochemical studies conducted with the use of heavy metals on plant cells translate into the heavy metal tolerance of the species.

## 1. Introduction

Since chemical and physical properties of essential and non-essential elements are similar, non-essential elements, which include some heavy metals, e.g., Pb, Cd, and Cr, enter plant cells through transporters for essential ions through the plasma membrane (‘by the way transport’) [1]. Plants have developed protective mechanisms against the toxic effects of non-essential metals and of increased amounts of essential heavy metals through their detoxification. This is achieved by different compounds (e.g., metallothioneins, phytochelatins, glutathione, and organic and amino acids) that bind heavy metals and ultimately transport and deposit heavy metals in the vacuole [2,3,4,5]. As long as the cell’s sequestration (binding or confining metal ions so that they are separated from other components of a biological system) proceeds well, the cell is protected against the detrimental effects of heavy metals. When sequestration fails, an oxidative burst and direct exposure to the toxic ions disrupt the cell membrane and damage its permeability, with proteins in the cytoplasm then being denatured. The secondary, tertiary, and quaternary structure of the protein is destroyed, and therefore, all levels of the three-dimensional structure of the protein molecule are violated, except for the primary structure. Ultimately, the biological activity of the proteins is destroyed, and the cell dies [6,7]. This kind of death, termed necrosis, differs at both the biochemical and ultrastructural levels from programmed cell death (PCD). Both could be the results of heavy metal action (for a revision, see Sychta et al., 2021 [8]). Suspended cells (derived from callus tissue) grown in in vitro culture reflecting many physiological and biological characteristics of the whole plant are a useful tool to study heavy metal toxicity and tolerance, including processes involved in their death [8,9,10,11].

Many species of the Violaceae Batsch. family show tolerance to increased concentrations of trace elements in the soil, mainly nickel, cadmium, zinc, and lead [12,13]. They belong to the *Viola*, *Hybanthus*, and *Rinorea* genera found in calamine, serpentine and arsenic-rich soils [14,15,16,17,18,19,20,21]. Some are hyperaccumulators of heavy metals, which means that they accumulate extremely large amounts of heavy metals in aboveground parts (e.g., *V. baoshanensis*, *V. yedoensis,* and *V*. *allchariensis*) [18,21,22]. Very little is known about mechanisms of heavy metal tolerance in violets. In the hyperaccumulator *V. baoshanensis*, enhanced degradation of misfolded proteins and up-regulation of genes related to sucrose metabolism and tonoplast transporters explain its high tolerance to heavy metals [23]. European members of the *Viola* genus are mostly facultative metallophytes (pseudometallophytes) with metallicolous and non-metallicolous populations formed at various mutual proportions (metallic populations predominate or non-metallic ones predominate, or there is a similar frequency of both population types). Less frequently, they are obligate metallophytes occurring exclusively on metalliferous soils, and are usually endemic [13,16,24,25]. As with hyperaccumulators, knowledge about the heavy metal tolerance mechanism and its acquisition in metallicolous populations is scarce. They must have developed or inherited efficient detoxifying mechanisms as their cell survivability after heavy metal treatment is high, and Zn and Pb are mostly deposited at the vacuole and also at the cell wall, thus out of the most sensitive protoplast of the cell [11].

Although tolerance to heavy metals seems to be a common phenomenon in *Viola* genus, it is unclear whether it is a result of microevolutionary processes that gradually occur and are a part of adaption to specific conditions or whether resistance was inherited from a metal-tolerant ancestor. Thus, adaptive tolerance would refer to the species whose individuals inhabiting only polluted areas possess this trait as a result of natural selection, whereas constitutive (innate) tolerance would refer to the species where all individuals (from polluted and non-polluted sites) manifest this feature. It has been confirmed that in some pseudometallophytes from other genera (e.g., *Arabidopsis halleri*, *Silene uniflora*) sufficient genetic variation allows populations to adapt quickly to a single physiological stress (heavy metal) repeatedly in different places [26,27]. However, multiple origins of metallicolous populations have never been confirmed in violets. It is only speculated that so-called ‘zinc violets’ (*V. lutea* ssp. *westfalica* and *V. lutea* ssp. *calaminaria*), which are descendants of the alpine *V. lutea*, growing in the past and nowadays in soil naturally enriched with heavy metals, could have inherited exceptional tolerance from their ancestor as their close relationship with *V. lutea* was revealed [28,29]. Although there are not too many reports, the possibility of creating heavy-metal-tolerant species through the hybridization process could not be excluded either, especially among easily hybridizing violets. Though not yet investigated for heavy metal tolerance, the introgressive form of *V. reichenbachiana* · *V. riviniana* seems to display high adaptation to heavy metal polluted sites in German and Polish populations [30,31,32].

The present study aimed to evaluate the hypothesis of innate (constitutive) tolerance of species belonging to the *Viola* genus. This was achieved by determining: (1) tolerance to Zn and Pb, (2) physiological response to heavy metals, and (3) strategy against heavy metals using cell suspension culture of several metallophytes and non-metallophytes from *Viola* and other genera. It was hypothesized that innate tolerance of *Viola* species should be independent of the metallophyte status of violets, and it also should be higher than in non-metallophyte from other genera *(A. thaliana*) and at least the same or even higher than in metallophyte from other genera (*S. vulgaris* subsp. *humilis*).

## 2. Materials and Methods

### 2.1. Plant Material

Five species of different metallophyte status were chosen: *Arabidopsis thaliana*—non-metallophyte (species intolerant to heavy metals), *Viola philippica*—Cd-hyperaccumulating metallophyte [21], *V.* x *wittrockiana*—metal-tolerant non-metallophyte which demonstrates tolerance to Zn and Pb in experimental conditions [9]; *V. tricolor*—facultative metallophyte, species occurring on both metalliferous and non-metalliferous soils, showing high tolerance to Zn and Pb [25], and *Silene vulgaris* subsp. *humilis*—facultative metallophyte [13]. The choice of species was dictated by the goal. We wanted to compare the tolerance level of violets with different metallophyte (and non-metallophyte) status with species from other genera (metal tolerant *S. vulgaris* subsp. *humilis* and intolerant *A. thaliana*).

Seeds of these five species came from various sources. *Arabidopsis thaliana* (L.) Heynh. Col-0 was supplied by the Arabidopsis Biological Resource Center, Ohio State University, Ohio, USA. Seeds of *Viola* *philippica* Cav. (*V. yedoensis* Makino) were collected in the vicinity of the Henan Institute of Science and Technology, Xinxiang, Henan province, China (35°27′50″ N, 113°46′02″ E) and of *S. vulgaris* subsp. *humilis* (Schubert) and *V. tricolor* L., in the old zinc-lead heap in Bukowno near Olkusz (Southern Poland, 19°25′96″ E, 50°17′30″ N). *Viola* × *wittrockiana* Gams. was purchased from a commercial supplier (POLAN, Poland).

### 2.2. Development of Tissue Culture on Solid Media

Seeds of all species were sterilized in 70% ethanol for 90 s and commercial bleach for 12 min and rinsed three times in sterile distilled water for 3, 7, and 10 min. Seeds were germinated on a solidified half-strength Murashige and Skoog (MS) medium [33] in sterile conditions for 2 weeks. Sterile leaves of the seedlings were placed on ½ MS media [33] or ½ MS with various combinations of auxins (2,4-dichlorophenoxyacetic acid; 2,4-D or α-naphthaleneacetic acid; NAA) and cytokinins (6-benzylaminopurine; BAP or kinetin; KIN) as follows: 2 mg L-1 2,4-D + 2 mg L^−1^ BAP, 2 mg L^−1^ 2,4-D + 2 mg L^−1^ KIN, 2 mg L^−1^ NAA + 2 mg L^−1^ BAP, 2 mg L^−1^ NAA + 2 mg L^−1^ KIN, 0.5 mg L^−1^ NAA + 5 mg L^−1^ BAP.

All media were supplemented with 30 g L^−1^ sucrose (Sigma-Aldrich, St Louis, MO, USA) and solidified with 8 g L^−1^ agar (Duchefa Biochemie, Amsterdam, The Netherlands). The pH of the medium was adjusted to 5.7–5.8. The cultivation was carried out under stable artificial conditions, in a growth chamber at 25 ± 3 °C with a 16 h photoperiod under cool-white-fluorescent lamps (light intensity 70–100 μmol m^−2^ s^−1^). Culture media and instruments were sterilized in a steam autoclave (121 °C, 1.05 bar; Prestige Medical, Lancashire, UK). The tissue cultures were passaged onto fresh media every 2–3 weeks. The callus tissue was initiated to develop after 1–3 weeks, depending on the species.

### 2.3. Development of Cell Suspension Cultures and Their Treatment with Heavy Metals

One gram of two-month-old callus tissue grown on leaf fragments cultivated on a solidified medium was fragmented and transferred into an Erlenmeyer flask (100 mL) containing 25 mL of liquid MS medium, supplemented with the same growth regulators as when obtaining callus tissue and 30 g L^−1^ sucrose. The flasks were kept on a rotary shaker (WL-972, JW Electronic, Warszawa, Poland) at constant speed (150 rpm) under conditions as described for the callus tissue culture. Suspension cultures were passaged and replenished with a fresh medium every two to three weeks. Ultimately, all suspensions were kept on the same medium (2 mg L^−1^ 2,4-D + 2 mg L^−1^ BAP) to standardize the culture conditions. To establish the growth kinetics of each cell suspension, 10 mL of cells from the suspension culture were mixed with 15 mL of the fresh medium, creating subcultures. Every third to fourth day, 1 mL of the subculture was taken and centrifuged at 1000 rpm, the supernatant was pulled away and the fresh weight of cells was measured. The experiment was repeated four times and the results were averaged.

Cell suspension cultures (25 mL in total) were treated with lead salt (Pb(NO_3_)_2_) or zinc salt (Zn(NO_3_)_2_ · 4H_2_O) during the exponential phase of culture growth at the following concentrations: 0 μM, 200 μM, 500 μM, and 1000 μM. Zinc and lead salts were added to the media and then the pH was adjusted to 5.7–5.8, because the addition of metal to the medium causes a significant decrease in pH [34]. Pb and Zn were chosen because they are the most common elements in calamine soils inhabited by violets and *S. vulgaris* subsp. *humilis*. Calamine soils are also rich in Cd, though its concentration is usually lower than Zn and Pb. The choice of cadmium hyperaccumulator not Zn or Pb was dictated by the fact that among violets there are no Pb hyperaccumulators at all, and those considered to be Zn hyperaccumulators turned out to be metal excluders [25]. Despite the formation of suspended insoluble salts and slight turbidity of the medium, after the addition of Pb to the cells, this element was available for plant cells as confirmed former experiments [34]. The amount of nitrates delivered altogether with heavy metals was irrelevant for the course of the experiment with heavy metals because in the medium there had been already a lot of nitrogen as its ingredient. Cell viability was estimated using the alamarBlue assay [11,34] at time 0 (before adding heavy metals) and after 24, 48, and 72 h. Selection of the Zn and Pb concentrations and time treatment was based on the rate of cell survival after heavy metal treatment. Too high a concentration caused cell death, and too low a concentration had no effect on the cells. Cell viability at time 0 was treated as 100%. Measurements were made in 3 biological and 3 technical replicates.

### 2.4. Quantification of Low Molecular Weight Organic Acids and Thiols

Cells treated with 200 μM of Zn or Pb for 72 h and non-treated were used for low molecular weight organic acids (LMWOAs) measurements. This concentration was chosen to assure survival of the less tolerant cells of *Arabidopsis thaliana*. Tartrate, malate, and citrate were quantified in water plant extracts using capillary electrophoresis coupled with a DAD (Agilent 7100 Capillary Electrophoresis, Agilent Technologies, Santa Clara, CA, USA) according to the protocol reported by Dresler et al., 2014 [35]. Both reduced glutathione (GSH) and phytochelatins (PCs) were assayed using capillary electrophoresis (Agilent 7100 Capillary Electrophoresis, Agilent Technologies, Santa Clara, CA, USA) coupled with LED-induced fluorescence detection (Zetalif LED 450 nm, Picometrics Technologies SAS, Toulouse, France) after derivatization with monobromobimane (Merck, Darmstadt, Germany) following a method described by Dresler et al., 2019 [36].

### 2.5. Localization and Content of Heavy Metals

Cells treated with Zn or Pb at the same way as for quantification of low molecular weight organic acids and thiols were centrifuged in a density gradient (0%, 6%, 10%, 18%) of Ficoll PM 400 (Sigma-Aldrich, St Louis, MO, USA) in 3% sucrose at 130 rpm for 5 min. Fractionation of cells was necessary to separate living cells from dead. The fractionated viable cells were collected and mixed with 3% sucrose and centrifuged again at 1000 rpm for 10 min to remove the Ficoll supernatant. Then, viable cells were dried at 100 °C for 48 h, weighed, and flooded with 400 μL of a mixture (7:1, *v:v*) of HNO_3_ and HClO_4_ (both ThermoFisher Scientific, Waltham, MA, USA), and then mineralized for four days at high temperature starting from 50 °C and increasing up to 150 °C. The digest was concentrated by evaporation and then diluted with 4 mL 0.2% HNO_3_. A Perkin-Elmer AAnalyst 200 (ThermoFisher Scientific, Waltham, MA, USA) was used to measure Zn and Pb content. Verification of accuracy was performed by analyzing three blanks and two reference samples. Pine needles 1572a [37] were used as standard material in the measurement of heavy metal concentrations. There were 5 replicates per treatment.

Leadmium™ Green AM Dye for Intracellular Detection of Lead and Cadmium (ThermoFisher Scientific, Waltham, MA, USA) was used according to the manufacturer’s protocol with some modification for the plant cell to visualize the distribution of lead within the cells. The Green AM stock solution was diluted in 3% sucrose (1:10, *v:v*) to prepare the working solution. Cells were centrifuged (1000 rpm), washed and resuspended in 3% sucrose. Then, 4 µL of Green AM working solution was added to 1 mL of cells and incubated at room temperature for 30 min. After centrifugation (1000 rpm) and washing with 3% sucrose, cells were observed and documented under a Nikon Eclipse E400 epifluorescence microscope (Nikon, Tokyo, Japan) (excitation λ = 490 nm, emission λ = 520 nm) equipped with a digital camera. Non-stained cells served as a control. The images were processed with NIS-Elements Viewer Imaging and Photoshop version 23.2.2 software. 

### 2.6. Statistical Analysis

Statistical analyses were conducted in R [38] using ape [39], vegan [40] packages while figures were produced using the ggplot2 [41], the ggthemes [42] packages. Multiple analysis of variance (MANOVA) was performed to assess the influence of metal dose, time and species on cell viability. Results of *V. tricolor* (metallicolous population) cell viability after treatments were taken from Sychta et al., 2018 [11]. To determine the significance of differences in cell viability and differences in Zn or Pb content in cells between species, one-way ANOVA, followed by the Tukey HSD test, for a given treatment time and metal dose was performed. Determination of differences between metabolite content was carried out using the Kruskal–Wallis test, followed by Dunn’s *post hoc* test. Values below detection limit was assumed as 0 for statistical analysis. Principal coordinate analysis (PCoA) was applied to evaluate the influence of heavy metal treatment on metabolite content. The results of *post hoc* tests are available in Supplementary Materials.

## 3. Results

### 3.1. Cell Suspension Cultures Development

The callus of each species was induced on almost all the used media in the time of two to three weeks after placing the explants on the appropriate medium. The fastest growth for most species was observed on a half-strength MS medium with the addition of 2 mg L^−1^ 2.4-D + 2 mg L^−1^ BAP. The only exception was *Silene vulgaris* subsp. *humilis*, whose callus grew fastest on the half-strength MS medium with the addition of 0.5 mg L^−1^ NAA and 5.0 mg L^−1^ BAP. Stable suspension cultures were successfully produced from fragmented callus on a liquid medium with the same macro- and micro-elements and plant growth regulators as used for the induction of the callus. Ultimately, all suspensions were kept on the same medium to standardize the culture conditions.

**Figure 1:**
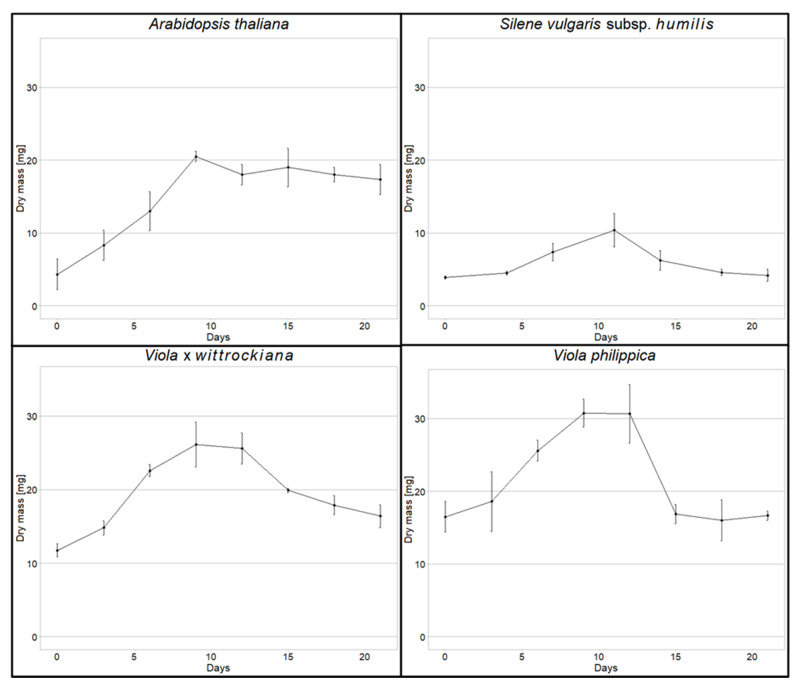
Plant cells growth in suspension monitored for 20 days. Each time point represents the mean value +/− SD of 4 measurements of dry weight. Growth curve of *V. tricolor* (metallicolous population) could be found in Sychta et al., 2018 [11].

At the starting point, the dry mass of the suspended cells of the species varied from 3.9 mg to 16.5 mg, depending on the species. Doubling of the mass was reached after 5–12 days. Logarithmic growth phase, at which cells were taken for heavy metal treatment, lasted even up to the 11th day, depending on the species (Figure 1).

### 3.2. Cell Viability after Heavy Metal Treatment

**Figure 2:**
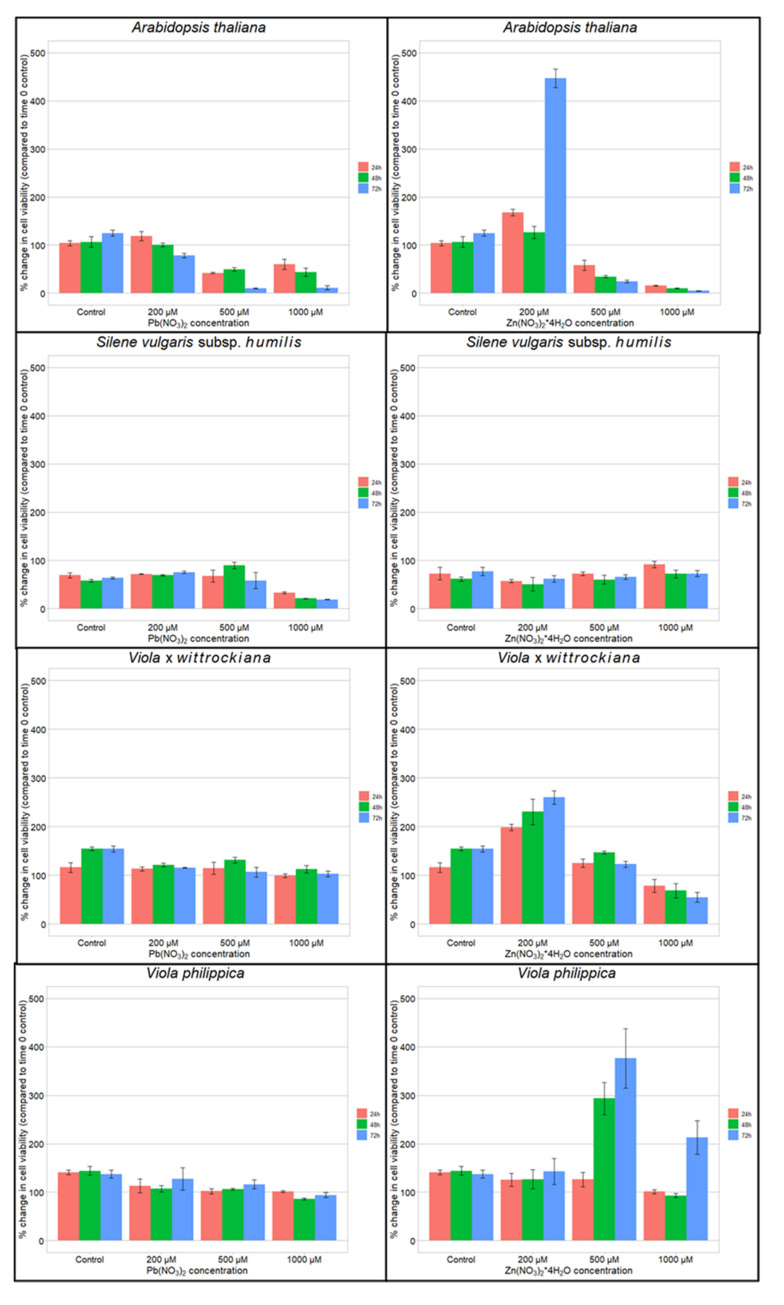
The mean frequency (+/−SD) of viable cells after treatment with different concentrations of Pb and Zn. Means and standard deviations were established based on altogether N = 9 measurements. Frequency of viable cells of *V. tricolor* (metallicolous population) after treatments could be found in Sychta et al., 2018 [11].

Cell viability at different time treatments and concentrations of Zn and Pb varied between species (Figure 2, Supplementary Tables S1–S3). Multivariate ANOVA showed differences between species depending on the concentration, and type of metal added. Under lead treatment, differences in time were significant (Supplementary Table S1). The cell viability of metallophytes—*S. vulgaris* subsp. *humilis*, *V. tricolor*, and the non-metallophyte—*V*. · *wittrockiana* treated with the highest (1000 μM) Zn concentration after 72 h (72.7%, 82.0%, 54.8%, respectively; Figure 2F,G) did not differ significantly (Supplementary Table S2, Tukey’s test, *p* > 0.05). Cells of *V.* x *wittrockiana*, *V. tricolor,* and *S. vulgaris* subsp. *humilis* showed high tolerance to Zn, similarly to cells of *V. philippica* (54.8%, 82.0%, 72.7%, respectively vs. 213.2%; 1000 μM; 72 h). *Arabidopsis thaliana* showed the lowest tolerance to Zn (4.7%; 1000 μM; 72 h).

The viability of cells of violets at the highest (1000 μM) lead concentration after 72 h (*V*. · *wittrockiana*: 94.0%, *V. philippica*: 102.9%; Figure 2C,D) did not differ significantly between each other (Supplementary Table S3, Tukey’s test, *p* > 0.05). Cells of all selected *Viola* species showed high tolerance to Pb (*V*. · *wittrockiana*, *V*. *tricolor*, *V. philippica*, 102.9%, 67.3%, 94.0%; 1000 µM, 72 h; respectively). *Arabidopsis thaliana* and *S*. *vulgaris* subsp. *humilis* showed very low tolerance to lead (11.3%, 18.9%; 1000 μM, 72 h; respectively) (Figure 2, Supplementary Table S3).

A hormesis effect (increase in the number of viable cells after heavy metal treatment in comparison with the control) was observed in *A. thaliana* after treatment with 200 µM Zn (Figure 2E) and in *V. philippica* after treatment with 500 µM Zn (Figure 2G). Ultimately, for each species cell viability decreased with increasing concentration and duration of heavy metal treatment (Figure 2, Supplementary Tables S2 and S3).

### 3.3. Non-Enzymatic Antioxidants of the Cells after Heavy Metal Treatment

**Figure 3:**
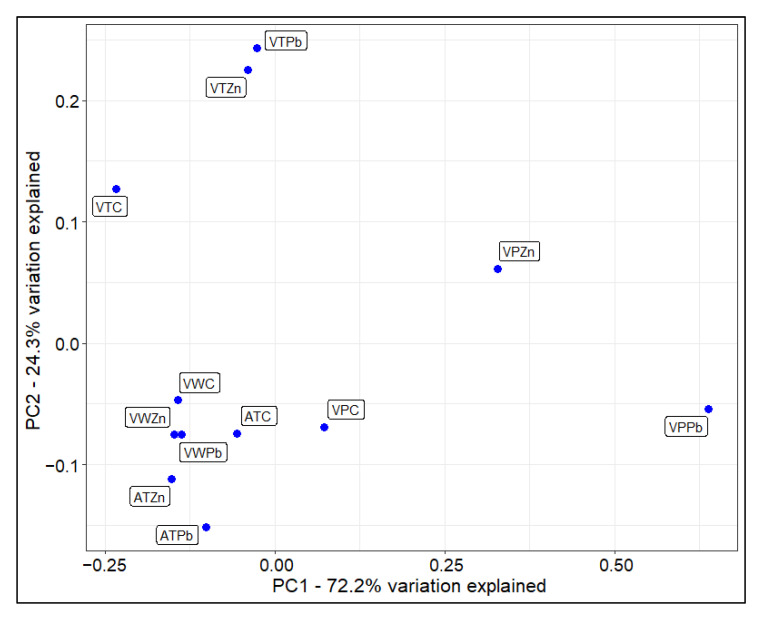
Principal coordinates analysis (PCoA) of Gower’s distances showing differences in average contents of non-enzymatic antioxidants of analyzed species after Zn or Pb treatments. AT—*Arabidopsis thaliana*; VW—*Viola* · *wittrockiana*; VT—*V. tricolor*; VP—*V. philippica*; C, Pb, Zn—control, lead, and zinc treatment, respectively.

In most species examined, the content of allantonin (ALLA) and ascorbic acid (L-AA) did not change after metal treatment, except for a decrease of L-AA in *A. thaliana* (both treatments) in comparison to the control treatment, in *V.* x *wittrockiana* (Zn treatment), a decrease to the control treatment of ALLA in *A. thaliana* (Zn treatment) and an increase to the control treatment of ALLA in *V. philippica* (Pb treatment) (Supplementary Table S4, Dunn’s test, *p* < 0.05). There was a significant increase to the control treatment of GSH in *V. philippica* (Zn treatment) and a significant decrease to the control treatment in *V. tricolor* (Pb treatment), and *A. thaliana* (Zn treatment) (Supplementary Table S4, Dunn’s test, *p* < 0.05). In all violets, the GSH content was much higher than in *A. thaliana*, and *V. tricolor* produced more GSH than other violets (Supplementary Table S5, Dunn’s test, *p* < 0.05). PCs, which were not detected in the control, were produced in all species after Zn or Pb treatment. PC3 was only detected in violets (very high increase in *V. philippica* after Pb treatment). PC4 occurred only in *V. philippica* after Pb treatment (Table 1). A significant increase to the control treatment in the content of tartrate and malate was observed in *V. philippica* and *V. tricolor* (Supplementary Table S4, Dunn’s test, *p* < 0.05). The citrate content increased significantly in comparison to the control treatment in *A. thaliana*, *V.* x *wittrockiana* and *V. philippica* after Pb treatment (Supplementary Table S4, Dunn’s test, *p* < 0.05). PCoA analysis showed the influence of heavy metal treatment on the metabolite content, particularly in *V. tricolor* and *V. philippica* cells (Figure 3). The distance between non-treated and heavy-metal-treated samples were the largest among all studied species.

### 3.4. Accumulation of Heavy Metals by Cells

**Figure 4:**
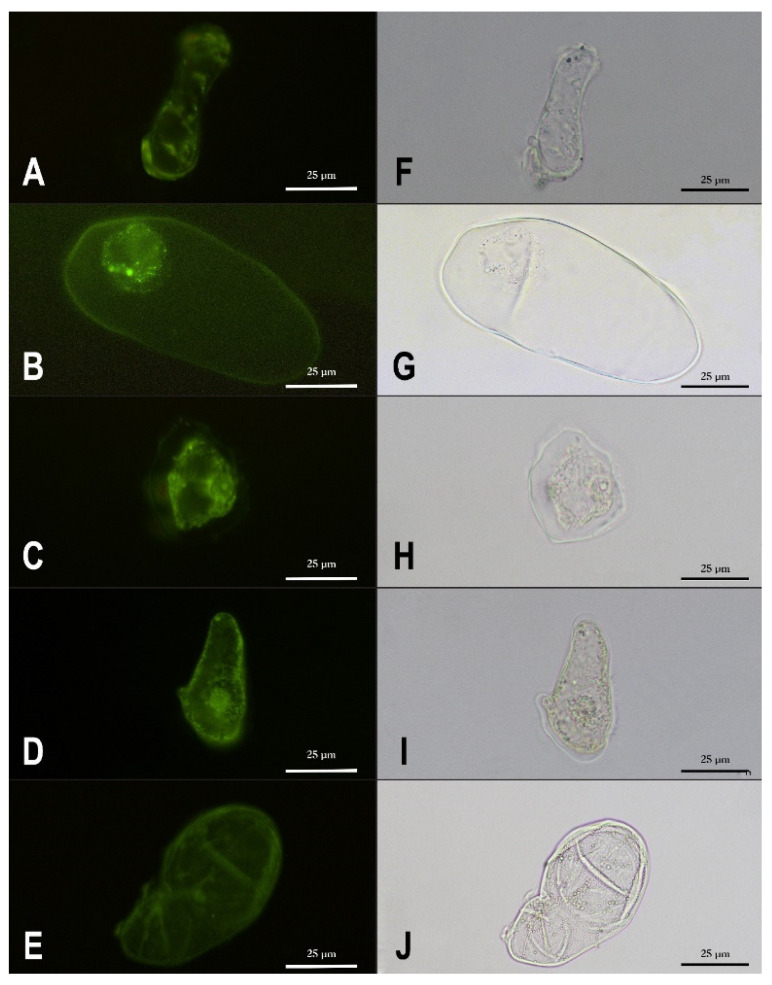
Intracellular lead detection after exposure to 200 µM of Pb using Leadmium Green staining. Left column: fluorescence microscopy; right column: light microscopy; *Arabidopsis thaliana* (**A**,**F**), *Silene vulgaris* subsp. *humilis* (**B**,**G**), *Viola* · *wittrockiana* (**C**,**H**), *V. tricolor* (**D**,**I**), and *V. philippica* (**E**,**J**).

**Figure 5:**
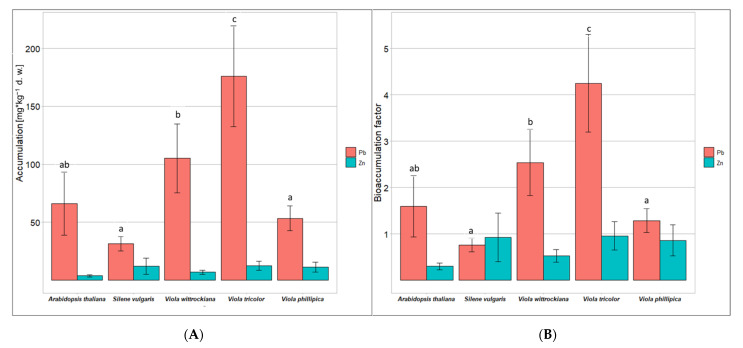
The mean Zn and Pb concentrations (**A**), and bioaccumulation factors [kg kg^−1^] (+/− SD) (**B**) in cell treated with 200 μM Zn or 200 μM Pb for 72 h. Different letters mean statistical differences (*p* ≤ 0.05) between species. Means and standard deviations were established based on altogether N = 5 measurements.

Although a large amount of lead was found in violets whose cells developed very thick cell walls, it seems that this element, when provided in moderate concentration (200 μM), was not accumulated within the cell wall (Figure 4C–E vs. Figure 4A,B and Figure 5A), in contrast to the cells of *A. thaliana* and *S. vulgaris* subsp. *humilis*. In all species, Pb was also accumulated at the protoplast of the cells, not in vacuoles (Figure 4). Confirmation that there was no lead in untreated cells is shown in supplemental materials (Supplementary Figure S1). The accumulation of Pb within the cells was approximately two to sixteen times higher, depending on the species, than the accumulation of Zn. Cells accumulated Pb in large amounts with significant differences between species (Figure 5A). *V. tricolor* had the highest lead concentration within its cells (Figure 5A). The differences in Zn accumulation between species were insignificant (Figure 5A). All species, except *S. vulgaris* subsp. *humilis*, revealed a bioaccumulation factor (the concentration of metal within cells/the concentration of metal in the medium) greater than 1 in lead treatment, indicating accumulation capacity. Such a strategy was not developed in the case of Zn treatment (Figure 5B).

## 4. Discussion

### 4.1. Cells of Viola Species Show High Tolerance to Pb and Zn

Undifferentiated and homogeneous cell suspensions growing under constant controlled conditions serve to study cell physiological processes, their reactions to abiotic and biotic stresses, genetic engineering, and secondary metabolism production [43,44,45]. In this study, we used them to compare the tolerance traits of violets in relation to the other species tolerant to heavy metals (*Silene vulgaris* subsp. *humilis*) and non-metallophytes (*Arabidopsis thaliana*, serving as a control). Our results show that all three violets, independently of their status (metal hyperaccumulator, metallophyte, and non-metallophyte), show higher tolerance to heavy metals than tolerant *S. vulgaris* subsp. *humilis* and intolerant *A. thaliana*. High tolerance of the cells of *Viola* · *wittrockiana*, a common bedding plant, is not an unexpected result. Sychta et al., 2020 [9] based on pot experiments measuring seed germination, heavy metal accumulation and sexual reproduction under the Zn and Pb treatments, confirmed that this non-metallicolous ornamental species could be treated as another metal-tolerant violet of similar tolerance to the metallicolous genotype of *V. tricolor*. Comparing our results with two accumulators *Jatropha curcas* and *Prosopis laevigata* [46,47], whose response to heavy metals varied depending on the metal applied, the dose and duration, is unauthorized neither due to the different methodology (biomass weight vs. cell viability) nor due to the fact that, in the latter biomass of all, not only living cells were measured. For heavy metals and metabolite content, we fractioned our cells to measure these parameters exclusively in living cells. Taking all of this into account, cells of violets show a high tolerance to Pb and Zn. Contrary to Pb that has no biological function, Zn is required by cells and has a well-defined biological function as a content of metalloenzymes [48]. Lead is also less mobile than zinc [34]. These two factors could explain the higher tolerance of cells of violets to Zn than to Pb.

### 4.2. Metallophyte Violets Produce High Content of Non-Enzymatic Antioxidants

The most important compounds that are ligands for metals involved in cellular, intercellular, and xylemic transport are GSH, PCs, histidine (His), and nicotinamine (NA). Organic acids are unlikely to form chelates in the cytoplasm, because the metal-acid bond is not stable at pH above 5. The main role of organic acids is metal sequestration rather than metal chelating, e.g., Fe^3+^ in the citrate complex [49]. We did not observe sequestered heavy metals in the vacuole; however, this could be due to the low resolution of light microscopy while Pb and Zn were localized in the vacuole of *V. tricolor* by an electron transmission microscope [11]. Considering that in *V. tricolor* and *V. philippica* under heavy metal treatment, some organic acids are increased, they could be responsible for the sequestration of Zn and Pb in these metallophyte violets. In the hyperaccumulating Zn (and accumulating Pb) ecotypes of *Sedum*, the ligand for both metals is GSH [50]. Our results show that, in violets, one of the main ligands could also be GSH. A high concentration of this metabolite is present in all three violets, hundreds of times more than in *A. thaliana*. PCs, which are GSH oligomers produced by the enzyme phytochelatin synthase, are considered as one of the elements of the normal, constitutional mechanisms of tolerance to non-essential metals, particularly Cd. They however are ligands not only for Cd but also for other metals, including Pb. The results of Schat et al., 2002 [51], studying a few hypertolerant species, did not provide evidence in favor of a role for PCs in the detoxification of essential metal micronutrients, such as Zn. This may explain why in respect of PCs production, violets, particularly the hyperaccumulator of Cd, *V. philippica* (increase from 0 to 142 nmol/g fresh mass), are responsive to non-essential Pb (not to an essential Zn). This is not the case of intolerant *A. thaliana* whose PCs production remains at 0 or a very low level (our studies) and *Silene vulgaris* after Pb treatment [51,52]. Semane et al., 2007 [53] confirmed that PCs synthesis, especially PC2, is enhanced in *A. thaliana* under Cd treatment. This is consistent with our results, as basically PC2 was produced in this species, however, its level was very low compared with *V. philippica.* Among the studied species, metallophyte violets, *V. philippica* and *V. tricolor,* are the most responsive species under Zn and Pb treatments. The content of their metabolites changes significantly after metal additions (Figure 3). Besides GSH and PCs, organic acids such as citrates and malates are likely to be the main antioxidants produced under heavy metal exposure in these violets.

### 4.3. Moderate Accumulation of Zn and Pb in Cells of Violets

Viable cells of all *Viola* species accumulated moderate amounts of Zn (6.88–12.51 mg kg^−1^ dry weight) and a high amount of Pb (53.33–175.97 mg kg^−1^ dry weight) at 200 μM. These values are much lower (220–360 and 180–450 times, respectively) than in cells of violets treated with 2000 μM Zn or Pb [11]. This means that the concentration applied is of paramount importance, as only a 10-fold lower concentration may reduce the accumulation capacity even by several hundred times in violets. It must be remembered that tolerance to heavy metals cannot be inferred from the levels of accumulation alone. Although cells of *A. thaliana* (non-metallophyte), *V.* x *wittrockiana* (metal tolerant non-metallophyte) accumulated roughly the same amount of lead as cells of *Silene vulgaris* subsp. *humilis* and *V. philippica* (both metallophyte) their viability was much higher. According to Audet and Charest 2007 [54], BF values above 1 indicate the potential of the species for hyperaccumulation. All violets and *A. thaliana*, even at moderate (200 μM) concentration of Pb revealed the ability to hyperaccumulate Pb; however, this ability was the strongest in *V. tricolor* and *V.* x *wittrockiana*, not in the hyperaccumulator of Cd—*V. philippica.* Despite the lower mobility and limited use (for plants) of lead compared to zinc, cells treated with the same amount of either Zn or Pb accumulated at least several times more Pb than Zn. Although this trend is not observed in the field, where violets accumulate more zinc than lead in roots and in the aboveground parts [25], high BF (above 4) was confirmed in *V. tricolor* treated (to the soil) with 10 and 100 ppm of Pb [9]. Similarly, high BF was also observed in *V*. · *wittrockiana* at low concentrations (10 ppm added to the soil) [9]. On the other hand, in the cells of violets treated with high concentrations of Zn or Pb (2000 μM), the trend was reversed, cells accumulated significantly higher (10×) amounts of lead than zinc and revealed a very high BF (>50) in respect to the former element [11]. This could be caused by the better bioavailability of Pb in the culture media [34] than in the soil condition. In our studies, lead was detected in the protoplast of the cells, not in the cell wall nor in the vacuole, which could be a result of the low Pb concentration applied. It seems that sequestration of Pb was limited. In other metal-tolerant plants, e.g., signal grass (*Brachiaria decumbens*), non-essential Pb was initially present in the cytoplasm of rhizodermal and cortical cells and was then sequestered in the cell wall [55].

### 4.4. Innate Tolerance to Heavy Metals of Viola Species

Our results combined with the results of Sychta et al., 2018 [11] show that cells of violets, despite representing different ecological statutes (non-metallophytes or facultative / obligate metallophytes), tolerate high concentrations of Pb and Zn, and accumulate high amounts of Pb. This tolerance seems to be higher than in other metallophyte species. One of the mechanisms of tolerance could be GSH synthesis, but there are other non-enzymatic antioxidants, such as PCs, malates, and citrates belonging to the defense mechanism. Future specific metabolome sequencing studies would shed light on the basis of this tolerance in violets. Overall, these results suggest that ancestral plasticity (here described as the ability to cope with heavy metals) could play an important role in adaptive parallel evolution in violets similarly to two independently evolved lineages of tolerant to zinc *Silene uniflora* [56].

It has been shown that two zinc violets (*V. lutea* subsp. *westfalica* and *V. lutea* subsp. *calaminaria*) originate from non-metallophyte, alpine species *V. lutea* [28,29]. Seedlings of the latter treated with Zn and Pb accumulate these metals in an amount similar to the obligate metallophyte *V. lutea* subsp. *westfalica*. Furthermore, in *V. lutea* under heavy metal treatment, the increase in biomass production is the highest among four violets studied [57]. Thus, both results suggest that *V. lutea*—the ancestor of zinc violets—could have been a metal-tolerant species. It is not rare that metal-tolerant species evolved from montane populations (for a revision, see Bothe and Słomka 2017 [13]) which could have been naturally affected by the increased amounts of heavy metals, typical for mountain soils [58]. Furthermore, the majority of European metallophytes of the Melanium section belonging to the *Viola* genus, e.g., *V. tricolor* investigated here, are commonly found in the mountains of the Balkan Peninsula [59,60]. This all suggests that a common ancestor of metal-tolerant violets could have grown in montane areas and may have been affected by the influence of the naturally enhanced concentration of heavy metals. It also cannot be ruled out that mycorrhiza is an important factor allowing violets to grow in heavy metal polluted soils. Almost all species studied in this regard develop this kind of symbiosis with endophytic fungi [16,25]. It has been known for a long time that mycorrhiza helps and increases tolerance to heavy metals. To sum up, though recent study has some constraints (e.g., limited number of species investigated and also physiological parameters) it could be concluded that violets are heavy-metal-tolerant species, and this trait could not be adaptive but rather inherited from the common ancestor(s). Mechanisms of their tolerance require further transcriptomic and metabolomic studies which are currently in progress.

## Figures and Tables

**Table 1:** The content of metabolites in fresh mass [±SD] of the cells after treatment with 200 μM lead or zinc.

Species	Treatment	ALLA mg/g	L-AA mg/g	GSH nmol/g	PC2 nmol/g	PC3 nmol/g	PC4 nmol/g	Tartrate mg/g	Malate mg/g	Citrate mg/g
*Arabidopsis thaliana*	0 μM	0.024 [±0.005]	0.293 [±0.089]	16.437 [±0.6335]	ND	ND	ND	0.024 [±0.002]	0.113 [±0.035]	0.007 [±0.002]
	200 μM Pb	0.024 [±0.003]	0.108 [±0.064]	8.216 [±1.2716]	3.741 [±0.607]	ND	ND	0.027 [±0.002]	0.177 [±0.029]	0.083 [±0.050]
	200 μM Zn	0.017 [±0.004]	0.096 [±0.062]	5.330 [±0.4109]	3.377 [±1.086]	ND	ND	0.027 [±0.006]	0.131 [±0.021]	0.036 [±0.023]
*Viola* · *wittrockiana*	0 μM	0.015 [±0.001]	0.124 [±0.021]	173.353 [±63.9950]	ND	ND	ND	0.024 [±0.003]	0.057 [±0.020]	0.001 [±0.000]
	200 μM Pb	0.018 [±0.002]	0.075 [±0.026]	203.789 [±16.6196]	3.246 [±0.694]	2.098 [±0.150]	ND	0.021 [±0.002]	0.083 [±0.032]	0.005 [±0.001]
	200 μM Zn	0.016 [±0.004]	0.045 [±0.015]	212.254 [±6.8863]	3.649 [±0.207]	ND	ND	0.022 [±0.005]	0.108 [±0.084]	0.003 [±0.001]
*V. tricolor*	0 μM	ND	0.133 [±0.052]	479.745 [±7.0620]	ND	ND	ND	0.025 [±0.001]	0.006 [±0.001]	0.020 [±0.004]
	200 μM Pb	ND	0.316 [±0.025]	407.607 [±25.1181]	2.169 [±0.478]	ND	ND	0.075 [±0.062]	0.012 [±0.003]	0.038 [±0.009]
	200 μM Zn	ND	0.293 [±0.033]	432.550 [±13.3967]	5.386 [±1.317]	2.312 [±0.279]	ND	0.065 [±0.039]	0.013 [±0.001]	0.055 [±0.035]
*V. philippica*	0 μM	0.019 [±0.001]	0.214 [±0.069]	202.427 [±31.9216]	ND	ND	ND	0.045 [±0.002]	0.431 [±0.068]	0.465 [±0.051]
	200 μM Pb	0.025 [±0.003]	0.290 [±0.050]	234.810 [±86.1177]	82.744 [±99.973]	141.209 [±82.618]	63.54 [±31.060]	0.083 [±0.012]	0.696 [±0.151]	1.046 [±0.224]
	200 μM Zn	0.023 [±0.003]	0.279 [±0.059]	342.980 [±28.3279]	56.363 [±14.759]	13.034 [±1.775]	ND	0.075 [±0.013]	0.678 [±0.217]	0.751 [±0.204]

ALLA—allantoin; L-AA—ascorbic acid; GSH—glutathione; PC2-4—phytochelatins 2–4; ND—not detected, N = 5.

## Data Availability

The data presented in this study are available on request from the corresponding author.

## Supplementary Materials

The following supporting information can be downloaded at: https://www.mdpi.com/article/10.3390/cells11152355/s1, Table S1: Three-way ANOVA results for effect of the species *(Arabidopsis thaliana, Silene vulgaris* subsp. *humilis, Viola* · *wittrockiana, V. tricolor* [11], and *V. philippica*); Table S2: Tukey’s HSD test results for effect of species (*Arabidopsis thaliana, Silene vulgaris* subsp. *humilis, Viola* · *wittrockiana, V. tricolor* [11], and *V. philippica*) on cell viability for zinc in each concentration and time combination; Table S3: Tukey’s HSD test results for effect of species (*Arabidopsis thaliana, Silene vulgaris* subsp. *humilis, Viola* · *wittrockiana, V. tricolor* [11] and *V. philippica*) on cell viability for lead in each concentration and time combination; Table S4: Dunn’s test results for effect of treatment (0, 200 μM lead, 200 μM zinc) on metabolite content in cells of *Arabidopsis thaliana, Viola* · *wittrockiana, V. tricolor,* and *V. philippica*). Table S5: Dunn’s test results for effect of species (*Arabidopsis thaliana, Viola* · *wittrockiana, V. tricolor,* and *V. philippica*) on metabolite contents in cell suspension culture for each treatment (0, 200 μM lead, 200 μM zinc); Figure S1: Intracellular lead detection after exposure to 200 µM of Pb using Leadmium Green staining (fluorescence microscopy).
