# Supplementary material for: Innate, High Tolerance to Zinc and Lead in Violets Confirmed at the Suspended Cell Level
Source: Cells. 2022 Jul 31;11(15):2355. doi: 10.3390/cells11152355 (PMC9367367; doi:10.3390/cells11152355)
Supplement: Supplementary file 1 [file cells-11-02355-s001.zip › cells-1783743-SM.pdf]

Table S1. Three-way ANOVA results for effect of the species (Arabidopsis thaliana, Silene vulgaris subsp. humilis, Viola x wittrockiana, V. tricolor, Sychta et al 2018 [11], and V. philippica).

	d.f.	MS	F-value	P-value
Zinc treatment				
Species	4	76730	142.38	0.00000
Time	2	24133	44.78	0.00000
Concentration	3	35138	65.20	0.00000
Species*Time	8	10429	19.35	0.00000
Species*Concentration	12	37397	69.39	0.00000
Time*Concentration	6	5595	10.38	0.00000
Species*Time*Concentration	24	9612	17.84	0.00000
Error	120	539		
Lead treatment				
Species	4	30281	370.23	0.00000
Time	2	121	1.47	0.232
Concentration	3	16986	207.68	0.00000
Species*Time	8	859	10.50	0.00000
Species*Concentration	12	2542	31.08	0.00000
Time*Concentration	6	616	7.52	0.00000
Species*Time*Concentration	24	348	4.26	0.00000
Error	120	82		

Concentration (0 μM, 200 μM, 500 μM, 1000 μM) and time of treatment (24 h, 48 h, 72 h) zinc and lead on cell viability. d.f., degrees of freedom; MS, mean square; F-value, value of F distribution; P-value, probability value.

Table S2. Tukey's HSD test results for effect of species (Arabidopsis thaliana, Silene vulgaris subsp. humilis, Viola x wittrockiana, V. tricolor, Sychta et al 2018 [11], and V. philippica) on cell viability for zinc in each concentration and time combination.

Concentration [μM]	Time [h]	Species				
		A. thaliana	*S. vulgaris* subsp. *humilis*	*V. x wittrockiana*	*V. tricolor*	*V. philippica*
0	24	104.1^{BC}	73.0^{A}	116.0^{CD}	88.5^{AB}	140.7^{D}
	48	106.6^{B}	61.8^{A}	154.1^{C}	90.3^{B}	144.2^{C}
	72	124.9^{B}	77.4^{A}	153.9^{C}	74.8^{A}	137.3^{B}
200	24	168.0^{BC}	57.6^{A}	198.4^{C}	67.6^{A}	125.2^{B}
	48	126.4^{B}	50.8^{A}	230.1^{C}	51.5^{A}	126.8^{B}
	72	446.8^{D}	62.1^{A}	259.7^{C}	47.2^{A}	143.1^{B}
500	24	57.7^{A}	72.4^{A}	124.9^{B}	55.2^{A}	126.1^{B}
	48	34.3^{A}	60.4^{A}	146.9^{B}	45.6^{A}	293.5^{C}
	72	24.5^{A}	65.5^{A}	122.5^{A}	43.1^{A}	376.4^{B}
1000	24	15.5^{A}	92.0^{B}	78.1^{B}	85.6^{B}	101.2^{B}
	48	9.9^{A}	72.1^{BC}	68.5^{B}	76.3^{BC}	93.0^{C}
	72	4.7^{A}	72.7^{B}	54.8^{B}	82.0^{B}	213.2^{C}

Different letters indicate significant differences ($p<0.05$).

Table S3. Tukey's HSD test results for effect of species (Arabidopsis thaliana, Silene vulgaris subsp. humilis, Viola x wittrockiana, V. tricolor Sychta et al 2018 [11], and V. philippica) on cell viability for lead in each concentration and time combination.

Concentration [μM]	Time [h]	Species				
		A. thaliana	*S. vulgaris* subsp. *humilis*	*V. x wittrockiana*	*V. tricolor*	*V. philippica*
0	24	104.1^{B}	69.4^{A}	116.0^{B}	88.5^{AB}	140.7^{C}
	48	106.6^{B}	58.1^{A}	154.1^{C}	90.3^{B}	144.2^{C}
	72	124.9^{B}	63.8^{A}	153.9^{C}	74.8^{A}	137.3^{BC}
200	24	118.8^{B}	72.0^{A}	113.1^{B}	65.7^{A}	113.0^{B}
	48	100.6^{C}	69.3^{B}	120.7^{D}	45.6^{A}	107.1^{CD}
	72	78.0^{AB}	75.9^{A}	115.5^{B}	75.5^{A}	127.1^{B}
500	24	42.0^{A}	67.8^{B}	114.4^{C}	67.4^{B}	102.0^{C}
	48	49.4^{A}	89.8^{B}	131.2^{C}	53.2^{A}	106.1^{B}
	72	10.0^{A}	58.4^{B}	106.7^{C}	64.3^{B}	116.2^{C}
1000	24	59.8^{B}	33.4^{A}	99.2^{C}	57.8^{B}	101.5^{C}
	48	43.7^{B}	21.1^{A}	112.6^{D}	42.7^{B}	86.2^{C}
	72	11.3^{A}	18.9^{A}	102.9^{C}	67.3^{B}	94.0^{C}

Different letters indicate significant differences ($p<0.05$).

Table S4. Dunn's test results for effect of treatment (0, 200 μM lead, 200 μM zinc) on metabolite content in cells of Arabidopsis thaliana, Viola x wittrockiana, V. tricolor, and V. philippica).

Metabolite	Species	Treatment [μM]		
		0	200 Pb	200 Zn
Alla	*A. thaliana*	0.024^{AB}	0.024^{A}	0.017^{B}
	V. x wittrockiana	0.015^{A}	0.018^{A}	0.016^{A}
	V. tricolor	ND	ND	ND
	V. philippica	0.019^{A}	0.025^{B}	0.023^{AB}
L-AA	*A. thaliana*	0.293^{A}	0.108^{B}	0.096^{B}
	V. x wittrockiana	0.124^{A}	0.075^{AB}	0.045^{B}
	V. tricolor	0.133$_{A}$	0.316 ^{B}	0.293 ^{B}
	V. philippica	0.214^{A}	0.290^{A}	0.279^{A}
GSH	*A. thaliana*	16.437$^{A`}$	8.216^{AB}	5.330^{B}
	V. x wittrockiana	173.353^{A}	203.789^{A}	212.254^{A}
	V. tricolor	479.745^{A}	407.607^{B}	432.550^{AB}
	V. philippica	202.427^{A}	234.810^{A}	342.980^{B}
PC2	*A. thaliana*	ND^{A}	3.741^{B}	3.377^{B}
	V. x wittrockiana	ND^{A}	3.246^{B}	3.649^{B}
	V. tricolor	ND^{A}	2.169^{AB}	5.386^{B}
	V. philippica	ND^{A}	82.744^{B}	56.363^{B}
PC3	*A. thaliana*	ND	ND	ND
	V. x wittrockiana	ND^{A}	2.098^{B}	ND^{A}
	V. tricolor	ND^{A}	ND^{A}	2.312^{B}
	V. philippica	ND^{A}	141.209^{B}	13.034^{AB}
PC4	*A. thaliana*	ND	ND	ND
	V. x wittrockiana	ND	ND	ND
	V. tricolor	ND	ND	ND
	V. philippica	ND^{A}	63.546^{B}	ND^{A}
Tartrate	*A. thaliana*	0.024^{A}	0.027^{A}	0.027^{A}
	V. x wittrockiana	0.024^{A}	0.021^{A}	0.022^{A}
	V. tricolor	0.025^{A}	0.075^{B}	0.065^{AB}
	V. philippica	0.045^{A}	0.083^{B}	0.075^{B}
Malate	*A. thaliana*	0.113^{A}	0.177^{B}	0.131^{AB}
	V. x wittrockiana	0.057^{A}	0.083^{A}	0.108^{A}
	V. tricolor	0.006^{A}	0.012^{B}	0.013^{B}
	V. philippica	0.431^{A}	0.696^{B}	0.678^{B}
Citrate	*A. thaliana*	0.007^{A}	0.083^{B}	0.036^{AB}
	V. philippica	0.465^{A}	1.046^{B}	0.751^{AB}
	V. x wittrockiana	0.001^{A}	0.005^{B}	0.003^{AB}
	V. tricolor	0.020^{A}	0.038^{AB}	0.055^{B}

Different letters indicate significant differences (p<0.05); ALLA – allantoin; L-AA – ascorbic acid; GSH – glutathione; PC2-4 – phytochelatins; ND – not detected. Not detected values were treated as 0.

Table S5. Dunn's test results for effect of species (Arabidopsis thaliana, Viola x wittrockiana, and V. tricolor, V. philippica) on metabolite contents in cell suspension culture for each treatment (0, 200 μM lead, 200 μM zinc).

Metabolite	Treatment [μM]	Species			
		A. thaliana	*V. x wittrockiana*	*V. tricolor*	*V. philippica*
ALLA	0	0.024^{A}	0.015^{AB}	ND^{B}	0.019^{A}
	200 Pb	0.024^{A}	0.018^{AB}	ND^{B}	0.025^{A}
	200 Zn	0.017^{A}	0.016^{AB}	ND^{B}	0.023^{A}
L-AA	0	0.293^{A}	0.124^{B}	0.133^{B}	0.214^{AB}
	200 Pb	0.108^{AB}	0.075^{B}	0.316^{C}	0.290^{AC}
	200 Zn	0.096^{AB}	0.0456^{B}	0.293^{A}	0.279^{AC}
GSH	0	16.437^{A}	173.353^{AC}	479.745^{B}	202.427^{BC}
	200 Pb	8.216^{A}	203.789^{AB}	407.607^{B}	234.810^{AB}
	200 Zn	5.330^{A}	212.254^{AC}	432.550^{B}	342.980^{BC}
PC2	0	ND	ND	ND	ND
	200 Pb	3.741^{AB}	3.246^{BC}	2.169^{C}	82.744^{A}
	200 Zn	3.377^{A}	3.649^{A}	5.386^{AB}	56.363^{B}
PC3	0	ND	ND	ND	ND
	200 Pb	ND^{A}	2.098^{AB}	ND^{A}	141.209^{B}
	200 Zn	ND^{A}	ND^{A}	2.312^{AB}	13.034^{B}
PC4	0	ND	ND	ND	ND
	200 Pb	ND^{A}	ND^{A}	ND^{A}	63.546^{B}
	200 Zn	ND	ND	ND	ND
Tartrate	0	0.024^{A}	0.024^{A}	0.025^{AB}	0.045^{B}
	200 Pb	0.027^{AB}	0.021^{B}	0.075^{A}	0.083^{A}
	200 Zn	0.027^{A}	0.022^{A}	0.065^{AB}	0.075^{B}
Malate	0	0.113^{AB}	0.057^{BC}	0.006^{C}	0.431^{A}
	200 Pb	0.177^{AB}	0.083^{BC}	0.012^{C}	0.696^{A}
	200 Zn	0.131^{AB}	0.108^{BC}	0.013^{C}	0.678^{A}
Citrate	0	0.007^{AB}	0.001^{B}	0.020^{AC}	0.465^{AC}
	200 Pb	0.083^{AB}	0.005^{C}	0.038^{BC}	1.046^{A}
	200 Zn	0.036^{AB}	0.003^{B}	0.055^{AB}	0.751^{A}

Different letters indicate significant differences (p<0.05); ALLA – allantoin; L-AA – ascorbic acid; GSH – glutathione; PC2-4 – phytochelatins; ND – not detected. Not detected values were treated as 0.

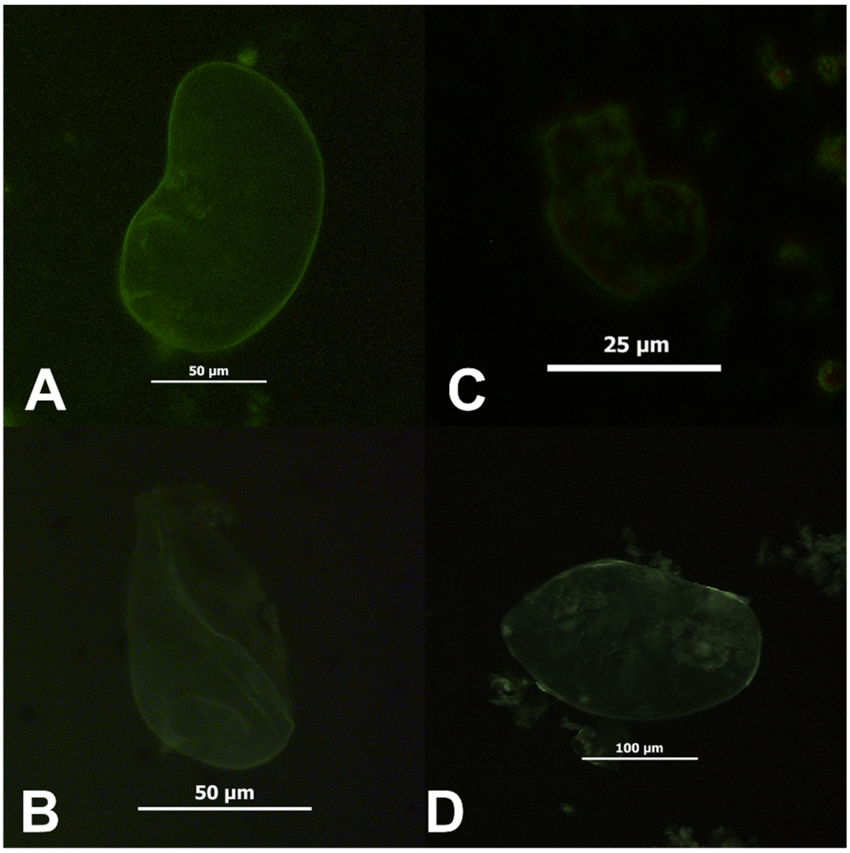

Figure S1. Intracellular lead detection after exposure to 200 µM of Pb using Leadmium Green staining (fluorescence microscopy); Silene vulgaris subsp. humilis (**A**), Viola x wittrockiana (**B**), V. tricolor (**C**), and V. philippica (**D**).
